# Skin Tape Stripping: A Non‐Invasive Approach Linking Epidermal Changes and Systemic Inflammation in Atopic Dermatitis

**DOI:** 10.1002/clt2.70179

**Published:** 2026-06-07

**Authors:** Ziyuan Tian, Ke Xue, Yong Cui

**Affiliations:** ^1^ Department of Dermatology China‐Japan Friendship Hospital Beijing China; ^2^ Graduate School Peking Union Medical College and Chinese Academy of Medical Sciences Beijing China

**Keywords:** atopic dermatitis, biomarkers, immunity, skin barrier, skin tape stripping

## Abstract

Atopic dermatitis (AD) is a complex chronic inflammatory skin disease driven by skin barrier dysfunction, immune dysregulation, and microbial imbalance. Traditional sampling methods, such as biopsies and blood collection, have provided valuable pathophysiological insights. However, their invasiveness, associated discomfort, and limitations for repeated sampling have constrained a dynamic understanding of disease progression and treatment responses. In recent years, skin tape stripping (STS) has emerged as a minimally invasive or even non‐invasive technique that addresses these limitations. STS enables the collection of corneocytes and upper granular layer cells from the epidermis, and when combined with high‐throughput multi‐omics technologies, such as RNA sequencing, proteomics, lipidomics, and metatranscriptomics, it provides a powerful platform to dissect the molecular mechanisms of AD, identify novel biomarkers, and facilitate stratification in precision medicine approaches.

## Graphical abstract

This review positions skin tape stripping as a methodological bridge in atopic dermatitis, linking epidermal molecular profiling with systemic inflammation. Integrated with multi‐omics technologies, STS enables non‐invasive biomarker discovery, disease endotyping, and treatment monitoring, advancing precision medicine for AD.

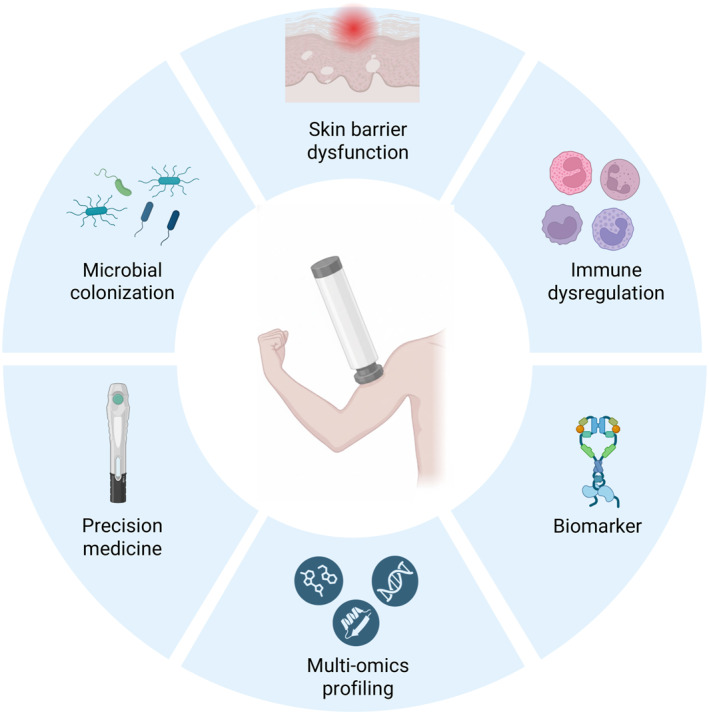

## 1 Introduction

Atopic dermatitis (AD) is a prevalent chronic inflammatory skin disorder characterized by intense pruritus, eczematous lesions, and recurrent flare‐ups [1]. The disease markedly impairs quality of life and is often accompanied by comorbidities such as sleep disturbances, anxiety, and depression [2]. The pathogenesis of AD is multifactorial and clinically heterogeneous, driven primarily by defects in skin barrier integrity and dysregulation of immune responses [2, 3, 4]. Although traditional methods, such as skin biopsies and blood analyses, have yielded valuable insights into these mechanisms, their invasive nature limits their broad clinical applicability. Recently, skin tape stripping (STS) has emerged as a non‐invasive and practical alternative that overcomes these limitations, providing a powerful tool and fresh perspective for elucidating the molecular basis of AD.

### 1.1 Pathogenesis of AD

The pathogenesis of AD centers on the dynamic interplay between skin barrier dysfunction and immune dysregulation [5]. Impaired barrier integrity and aberrant immune activation form a self‐perpetuating cycle: barrier disruption triggers inflammation, which subsequently exacerbates barrier damage. Furthermore, genetic predisposition, environmental exposures, microbial imbalance, and multiple immune axes—predominantly Th2, alongside Th1, Th17, and Th22 pathways—collectively shape the heterogeneous clinical manifestations of AD [3].

#### 1.1.1 Skin Barrier Dysfunction: A Core Driver

Skin barrier dysfunction is a central pathogenic process in AD, and some studies even suggest it precedes immune activation [6]. This comprehensive impairment involves both structural and functional abnormalities, leading to increased transepidermal water loss (TEWL) and heightened susceptibility to allergens, irritants, and microbial invasion [3].

At the molecular level, barrier breakdown is orchestrated by a synergistic triad of genetic, biochemical, and structural defects. Loss‐of‐function mutations in the filaggrin (FLG) gene form the major genetic basis of this defect [7]. These mutations weaken the mechanical integrity of the stratum corneum and lead to xerosis due to reduced production of natural moisturizing factors (NMFs) derived from FLG degradation [6, 8]. In parallel, the biochemical disruption of intercellular lipid homeostasis—specifically a reduction in total ceramide content and a shift toward shortened fatty acid chains—disturbs lamellar lipid organization and severely impairs the hydrophobic barrier [9, 10]. Furthermore, structural defects in tight junctions within the granular layer weaken the secondary barrier that regulates paracellular permeability [11]. Beyond these physical and chemical components, the skin microbiome constitutes a critical biological barrier. Microbiome dysbiosis—particularly overgrowth of *Staphylococcus aureus*—degrades epidermal junctions via secreted proteases and exacerbates local inflammation [12, 13].

Importantly, the exact molecular profile of this barrier dysfunction is highly heterogeneous across clinical subtypes. For example, patients with food allergy–associated AD (AD FA^+^) exhibit distinct alterations in epidermal architecture compared to those without food allergies [8]. Similarly, structural barrier phenotypes differ significantly between Type 2‐high and Type 2‐low inflammatory endotypes [14]. Therefore, precisely elucidating these localized, subtype‐specific barrier disruptions is crucial for understanding the diverse disease trajectories of AD and highlights the need for targeted epidermal profiling.

#### 1.1.2 Immune Dysregulation: The Cytokine Network

Immune dysregulation constitutes a central driver of AD, characterized by a predominant Th2‐type immune response embedded within a complex network also involving Th1, Th17, and Th22 pathways [15]. Historically, AD pathogenesis was framed as a dichotomy between barrier‐initiated and immune‐initiated mechanisms, but it is now widely recognized that the two processes act reciprocally and synergistically [16, 17].

In the barrier‐initiated cascade, epidermal damage induces keratinocytes to release alarmins including TSLP and IL‐33, which drive downstream immune activation [3]. In the immune‐initiated pathway, by contrast, intrinsic dysregulation leads to elevated IL‐4 and IL‐13^17^, which directly impair epidermal barrier integrity. Despite these distinct initiating routes, both pathways converge on Th2 axis activation—the hallmark of AD [18].

The key Th2 cytokines IL‐4 and IL‐13 exert dual pathogenic functions: they suppress FLG expression via STAT6 signaling [18], and promote inflammatory cell infiltration through chemokines such as TARC/CCL17 [19, 20, 21]. Additional immune axes further shape disease heterogeneity: Th1 activation dominates chronic lesions, while Th17 and Th22 signatures are particularly prominent in Asian and pediatric populations, collectively amplifying barrier damage and defining distinct AD endotypes [16, 22, 23].

#### 1.1.3 Pruritus, Microbiome Dysbiosis, and Environmental Factors

AD progression is further amplified by external and behavioral exacerbators. Building upon its role in biological barrier degradation, microbiome dysbiosis—dominated by *Staphylococcus aureus* overgrowth [8]—actively stimulates epidermal and immune cells to release pro‐inflammatory factors, directly amplifying the core Th2 immune response [12, 24, 25].

Concurrently, environmental pollutants further compromise barrier integrity and activate cutaneous inflammatory signaling via the aryl hydrocarbon receptor. This localized inflammation is further compounded by pruritus [26]. Driven by type 2 cytokines like IL‐31 acting on sensory neurons, intense itching provokes scratching that mechanically disrupts the barrier and releases additional inflammatory mediators. Together, these elements lock the patient into a self‐perpetuating itch‐scratch‐inflammation cycle that actively drives disease progression [4].

### 1.2 Traditional Sampling Methods

Traditional sampling approaches, including skin biopsies and blood collection, have provided fundamental insights into AD pathogenesis.

Skin biopsies are unique in providing simultaneous access to both the epidermis and dermis, enabling comprehensive evaluation of histological changes, cellular infiltration, and inflammatory responses within the deep dermis [14, 27, 28, 29]. Biopsies are particularly advantageous for investigating specific immune pathways, such as Th1 and Th22, and for detecting dermal cytokines (e.g., IFN‐γ) and epidermal proliferation markers (e.g., KRT16) [30, 31], offering novel insight into deep skin pathology [32]. However, their invasive nature poses significant limitations: they cause patient discomfort, may leave scars, and are poorly tolerated in pediatric populations, rendering frequent or longitudinal sampling impractical [21, 33]. Moreover, the pronounced spatial heterogeneity of AD lesions means that a single biopsy may not fully represent the molecular landscape of the affected area or capture dynamic changes across disease stages [34]. Furthermore, the coexistence of epidermis and dermis in biopsy samples can “dilute” signals from molecules predominantly expressed in the epidermis (such as FLG and loricrin), reducing detection sensitivity and potentially biasing assessments of functional protein levels [28, 30, 35].

In contrast, blood sampling offers a minimally invasive and convenient method, ideal for large‐scale or longitudinal studies. Blood reflects the systemic immune dysregulation characteristic of moderate‐to‐severe AD and provides a valuable window for assessing responses to systemic therapies [20, 36]. However, its systemic nature is also a limitation: blood cannot accurately capture local skin barrier defects or the intricate interactions between epidermal and immune cells. Key local inflammatory signals (such as innate immune activation and elevated IL‐1α and IL‐18) are often weak or undetectable in circulation [36, 37]. Therefore, blood biomarkers may respond too subtly or with delay to reflect the effects of topical treatments, and systemic drug effects in the skin and blood are not always temporally synchronized [38].

**FIGURE 1:**
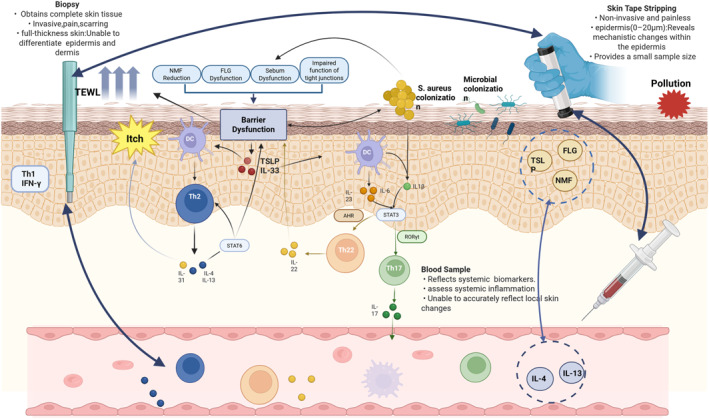
The pathophysiological landscape of atopic dermatitis and the complementary roles of different sampling modalities. The pathophysiological landscape of AD and the complementary roles of different sampling modalities. It presents the core mechanisms of AD, highlighting the interplay between barrier defects and immune pathways. Three key sampling methods are contrasted: skin tape stripping, a non‐invasive tool focused on the epidermis, is ideal for monitoring barrier integrity and superficial inflammation. A skin biopsy provides a complete, yet invasive, cross‐section of the skin, essential for studying deep dermal processes. Finally, blood sampling assesses the systemic dimension of AD by detecting circulating cytokines that reflect the overall inflammatory load. Together, these approaches enable a multi‐dimensional understanding of AD, from surface‐level changes to systemic immune status. AD, atopic dermatitis. Created with BioRender.com.

Given these limitations, STS has emerged as a non‐invasive alternative that bridges the gap between local and systemic perspectives. STS directly links epidermal pathophysiological changes with systemic inflammation, offering a more integrated view of disease biology [20, 36]. A truly comprehensive understanding of AD requires a multi‐modal sampling framework. Similar to conventional multi‐omics, which integrates different molecular layers (e.g., transcriptomics, proteomics), the strategic combination of sampling modalities represents a form of “methodological multi‐omics.” This approach, uniting the epidermal perspective from tape stripping, the dermal‐epidermal perspective from biopsies, and the systemic perspective from blood, is crucial for constructing a multi‐dimensional understanding of AD's pathophysiology [28, 30] (Figure 1).

### 1.3 STS: Technology and Advantages

#### 1.3.1 The STS Protocol: From Sample Collection to Extraction

STS is a non‐invasive technique that uses specialized tape to collect corneocytes and biomolecules from the epidermis, avoiding dermal contamination inherent to biopsies [39]. STS samples can be used for various downstream analyses, including RNA, protein, lipidomics, and microbiome studies [33]. Its simplicity, safety, pain‐free application, and repeatability make STS particularly suitable for pediatric populations and for longitudinal monitoring of dynamic changes in inflammatory skin diseases [33, 34, 40]. Various professional tapes are commercially available for STS; while sampling efficiency may vary, most are effective for successful biomolecule collection [41].

The core STS procedure involves repeated application and removal of the tape at the same site under consistent pressure, removing the epidermis layer by layer. The sampling depth is primarily determined by the number of sequential strips. For example, 35 D‐squame strips can fully remove the stratum corneum [41, 42]. The stable concentration of biomarkers across successive strips confirms the technique's high reliability and supports streamlined protocols [43].

Extraction protocols are tailored to the physicochemical properties of target analytes. For RNA, tape samples are initially treated with a strong lysis buffer, such as RNA lysis buffer, followed by purification using commercial kits. Due to the limited yield from a single strip [44], a pooling strategy—combining multiple strips—is commonly employed to enrich RNA content for downstream analysis [45]. The extracted RNA is remarkably stable, remaining suitable for sequencing for up to 3 days at room temperature, facilitating sample handling and logistics [46].

For transcriptomic profiling, data normalization relies on a dual‐stage framework. At the experimental stage, while full‐thickness tissue biopsies yield high amounts of RNA (∼4500 ng), STS samples typically yield only ∼22 ng, necessitating low‐input microamplification kits with strictly standardized RNA input (e.g., 5 ng) [28, 30]. Post‐sequencing, bioinformatic algorithms including DESeq2 (using the median‐of‐ratios method) and Limma‐voom are applied to mitigate biases from library size differences and support robust differential expression analysis [28, 30, 31].

Protein extraction is well established: the tape is typically immersed in a buffer (e.g., PBS or RIPA) containing detergents (e.g., Tween‐20) and protease inhibitors, with elution aided by physical disruption, including sonication [20, 21, 36]. For small, specialized proteins, such as antimicrobial peptides, chemical denaturation using 8 M urea can be used [47]. To ensure cross‐study comparability, biochemical normalization is indispensable. Following total protein quantification via BCA assay, researchers routinely normalize total protein input to a uniform, fixed baseline [8, 48]; for instance, a standardized input of 10 ng per sample is used for high‐sensitivity platforms including MSD and Olink [24, 49, 50, 51], to enable consistent, unbiased comparison of cytokine abundance. Similarly, for mass spectrometry‐based proteomic workflows, equal amounts of protein (e.g., 10 μg for TMT labeling) are processed in parallel across all samples to maintain biochemical consistency and analytical uniformity [8].

Recent methodological advances have expanded the analytical scope of STS beyond proteomic and transcriptomic profiling to encompass lipidomic and lipophilic hormone analyses [52]. In contrast to the aqueous buffer systems utilized for protein recovery, lipid extraction follows a straightforward, organic solvent‐based workflow: tape strips are typically eluted in methanol, vortexed, and stored at −20°C prior to downstream detection [52, 53].

For quantitative lipidomic analysis, robust normalization necessitates a dual‐assay quantification strategy. Initially, the absolute abundance of key barrier lipids is determined in picomoles (pmol) using high‐performance liquid chromatography‐tandem mass spectrometry (HPLC‐MS/MS) [10, 54]. To mitigate sampling variability and enable accurate cross‐cohort comparisons, these absolute lipid measurements are subsequently normalized against the total protein content derived from the identical tape strip, yielding a standardized index (e.g., pmol lipid/mg protein) [10].

**FIGURE 2:**
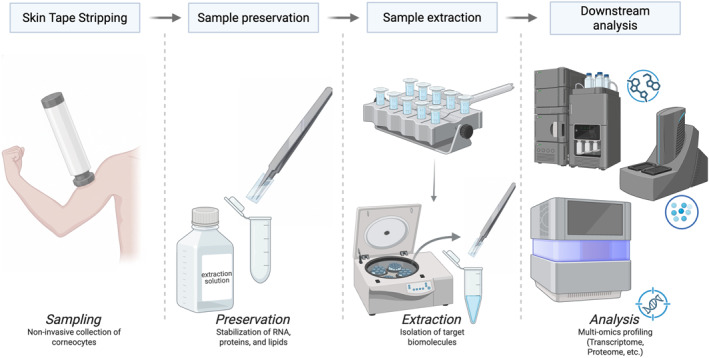
The skin tape stripping (STS) workflow for multi‐omics analysis. The skin tape stripping workflow for multi‐omics analysis. It outlines the standardized procedure for utilizing skin tape stripping as a powerful tool for molecular research. The workflow begins with sampling, where an adhesive tape non‐invasively collects corneocytes and biomolecules from the stratum corneum. Immediately following is preservation, where the tape is submerged in a solution to stabilize fragile molecules like RNA and proteins. In the extraction stage, target biomolecules are eluted and isolated from the adhesive matrix. Finally, the purified extract is subjected to downstream analysis using high‐throughput technologies, enabling comprehensive multi‐omics profiling including transcriptomics, proteomics, lipidomics, and microbiome analysis‐to provide deep molecular insights into skin health and disease. Created with BioRender.com.

The standardized STS workflow, from sample collection to downstream multi‐omics analysis, is illustrated in Figure 2.

#### 1.3.2 Synergy With Modern Analytical Technologies

Although STS yields relatively small amounts of biomolecules, the high sensitivity of modern analytical technologies fully compensates for this limitation. Next‐generation sequencing enables comprehensive profiling of gene expression in AD skin from minute samples, revealing dysregulated pathways, barrier gene defects, and microbiome activity. Similarly, high‐resolution mass spectrometry provides direct insight into barrier protein damage, inflammatory mediators, and lipid abnormalities, including ceramide deficiencies [8, 9, 49, 51, 55].

The combination of STS with advanced analytical techniques offers several key advantages. First, its non‐invasive and convenient nature minimizes the physiological and psychological burden on patients, especially children and other sensitive populations [50]. This method has been successfully used for dynamic monitoring of lesional and non‐lesional skin in pediatric AD patients, uncovering age‐specific molecular phenotypes without repeated biopsies or frequent blood draws [31, 56]. This capability opens new avenues for long‐term clinical monitoring and longitudinal studies. Second, STS enables precise characterization of the molecular heterogeneity of AD. Its simplicity allows multi‐site sampling to capture spatial and inter‐individual differences [57, 58]. Furthermore, STS can be applied to non‐lesional skin to detect early or subclinical molecular changes, providing critical insight into disease progression and potential recurrence [31]. Together, these features make STS a powerful platform for integrating molecular analysis with patient‐centered, minimally invasive sampling.

#### 1.3.3 Clinical Applications in Disease Management

By integrating proteomics, lipidomics, and sensitive immunoassays such as Multiple Analyte Profiling (MSD), STS provides a unique window into the core pathogenesis of AD. Studies consistently demonstrate elevated innate immune cytokines (IL‐1β, IL‐18, IL‐8) and Th2‐associated chemokines (TARC, CCL22) in tape strips from lesional AD skin [16, 37, 54].

Proteomic analyses of tape strip samples have revealed a cluster of 45 proteins (PC1) strongly correlated with TEWL and allergen polysensitization in pediatric patients with AD FA^+^, suggesting a distinct proteomic endotype [55]. Levels of NMF, a FLG breakdown product, are significantly reduced in tape strip samples from AD patients and correlate with both disease severity and treatment response [21, 29, 50]. Lipidomic studies have confirmed ceramide deficiencies in AD skin [59], and combined analyses of lipid profiles, family history, and type 2 cytokines have demonstrated strong predictive power for the risk of AD [54]. Interestingly, STS has also revealed AD‐like ceramide deficiencies in the healthy skin of pediatric patients with eosinophilic esophagitis, suggesting potential utility for monitoring internal disease states [10].

These molecular insights have direct clinical applications. In healthy infants, specific epidermal biomarkers captured by STS at 2 months of age can predict future AD onset. For instance, elevated expression of thymic stromal lymphopoietin (TSLP) in the stratum corneum is strongly associated with AD development by 24 months, with its predictive value further magnified when combined with a family history of atopy [60]. Similarly, elevated levels of Th2‐associated chemokines, such as TARC/CCL17, at 2 months also serve as robust early risk biomarkers for subsequent AD onset [61, 62]. In infants with established lesions, elevated IL‐2, CCL26, and CCL20 levels indicate a higher risk of AD progression, with IL‐2 acting as a key prognostic marker once inflammation is present [54, 63].

STS also enables sensitive monitoring of treatment responses. Proteomic studies have shown that dupilumab reduces 136 proteins, including immune and cardiovascular risk markers [51, 64]. Although both abrocitinib and dupilumab improve barrier function, they modulate distinct protein networks, with abrocitinib demonstrating superior restoration of key proteins such as FLG‐2 [64, 65]. Post‐JAK inhibitor treatment, some patients show clinical improvement while retaining elevated inflammatory proteins (e.g., TARC/CCL17), suggesting a risk of recurrence [49]. Furthermore, STS can distinguish the efficacy of different topical medications: betamethasone more effectively reduces skin cytokines, whereas tacrolimus primarily improves skin hydration [50].

STS also illuminates site‐specific differences in therapeutic response. Cytokine expression varies across lesional anatomical sites: for example, forehead lesions exhibit higher Th1/Th17‐associated factor levels, which may explain their slower resolution with IL‐4 receptor inhibitors [58]. Beyond these localized variations, STS multi‐omics holds potential for systemic AD endotyping to inform precision therapy, particularly for distinguishing Type 2‐high and Type 2‐low inflammatory signatures. Transcriptomic profiling of STS samples has identified the Type 2‐high endotype (defined by upregulated IL‐13 and IL‐4R) in multiple cohorts, with this signature reported to correlate with clinical disease severity in the studied populations [14]. While STS is widely used to monitor post‐treatment response, baseline STS molecular profiling may also inform upfront biologic selection. Prospective stratification with this approach could optimize treatment outcomes: patients with a dominant Type 2‐high baseline signature may respond better to targeted therapies like dupilumab, while those with mixed inflammatory signatures may derive greater benefit from broader‐acting agents such as JAK inhibitors [49, 64].

STS‐derived biomarker levels also correlate with disease activity: cytokines including TARC, CTACK, IL‐8 and IL‐18 have been linked to SCORAD (Scoring Atopic Dermatitis) and TEWL [16, 19]. Moreover, NMF and cytokine profiles differ in patients with FLG mutations and food allergies, suggesting STS may surpass biopsies in detecting these specific biomarkers [48].

STS also supports differential diagnosis. RNA sequencing of tape strip samples can distinguish AD from psoriasis through unique immune signatures and NOS2/iNOS expression [66]. It can differentiate types of hand eczema (allergic vs. irritant contact dermatitis) and characterize chronic hand eczema subtypes with or without AD [67, 68].

Collectively, these findings confirm STS is a powerful tool, with significant potential in disease prediction, prognosis, treatment monitoring, severity assessment, and differential diagnosis in AD (Table 1).

**TABLE 1:** Applications and discoveries of skin tape stripping combined with multi‐omics technologies in AD research.

Research area	Technology used	Key findings
Skin barrier function	Proteomics	— Identified a protein cluster (PC1) specific to AD FA+, associated with TEWL and allergen polysensitization — NMF levels correlate with AD severity and treatment response — Revealed differences in barrier protein repair mechanisms between abrocitinib and dupilumab
	Lipidomics	— Revealed disease‐specific lipid‐microbe correlations in AD skin (e.g., *S. hominis* and ceramides) — Identified unique lipid deficiencies in AD FA+ patients
Immune pathways & cytokines	Proteomics/cytokine profiling	— Detected elevated inflammatory markers in AD skin (e.g., IL‐1β, IL‐18, CCL17, etc.) — Revealed immune signatures of AD patients across different age groups — Monitored immunological changes following treatments (e.g., dupilumab, JAK inhibitors)
Microbiome	Metagenomics/16S rRNA	— Identified increased *S. aureus* abundance in non‐lesional skin of AD FA+ — Revealed complex interactions between *S. hominis* and host lipid metabolism
	Metatranscriptomics	— Identified active microbial species and their functions — Provided more dynamic functional information than genomics
Novel biomarkers	Proteomics/cytokine profiling	— Elevated skin TARC/CCL17 levels in infancy can predict AD onset — Identified a unique proteomic endotype for AD FA+ — Identified immune markers for monitoring treatment response
	RNA sequencing	— Differentiated unique immune and barrier features of AD from psoriasis and hand eczema — Assessed biomarkers related to disease severity, *FLG* mutations, and food allergies

Abbreviations: 16S rRNA, 16S Ribosomal RNA; AD FA+, Food‐Allergy‐Associated AD; AD, Atopic Dermatitis; CCL17, Chemokine (C‐C motif) Ligand 17; FLG, Filaggrin; IL, Interleukin; JAK, Janus Kinase; NMF, Natural Moisturizing Factor; PC1, Protein Cluster 1; RNA, Ribonucleic Acid; *S. aureus*, *Staphylococcus aureus*; *S. hominis*, *Staphylococcus hominis*; TARC, Thymus and Activation‐Regulated Chemokine; TEWL, Transepidermal Water Loss.

#### 1.3.4 Limitations of STS: Why a Multi‐Modal Bridge Is Indispensable

While STS provides a non‐invasive vantage point for interrogating the epidermal microenvironment, it is inherently constrained by several technical and biological limitations that restrict its utility when used in isolation.

The primary physical constraint is sampling depth; STS exclusively collects corneocytes and cells from the upper granular layer. While this superficial sampling avoids dermal interference, it fundamentally cannot capture key dermal pathological features or the deep Th1 immune axis prominent in adult chronic AD, representing a significant gap in clinical translation depth [20, 35]. Furthermore, STS is currently limited by substantial biological heterogeneity and an absence of standardized sampling protocols. Variations in stratum corneum thickness at different anatomical sites, inconsistent applied pressure, and demographic factors including age and ethnicity can all markedly influence biomarker yields [42]. Importantly, the broader generalizability of STS‐based endotyping requires cautious interpretation. To date, foundational data have been generated primarily from restricted demographic cohorts, predominantly adult Caucasian populations [37, 50, 58]. Indeed, epidermal biomarker profiles have been observed to differ markedly across diverse populations [43]: Asian and pediatric cohorts [48], for example, often present with prominent Th17 and Th22 signatures concurrent with Th2 activation [16], a pattern aligned with a mixed inflammatory phenotype or Type 2‐low‐predominant endotype. Such inherent variability renders it highly challenging to define standardized reference intervals or to carry out robust, objective cross‐center comparisons [42, 43, 48, 58].

**FIGURE 3:**
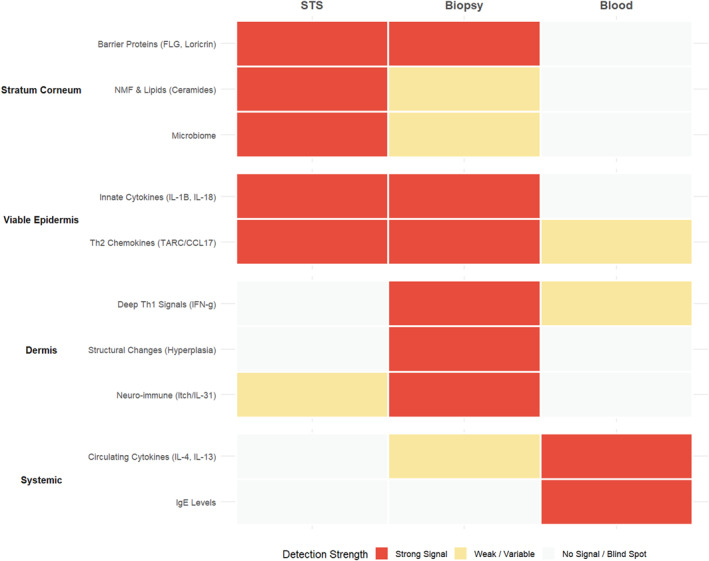
Heatmap comparison of signal detection depth across sampling modalities in atopic dermatitis. This figure compares the detection sensitivity of Skin Tape Stripping (STS), skin biopsy, and blood across biological layers from the skin surface to systemic circulation. Red indicates strong signal capture with high sensitivity and specificity. Yellow indicates weak or variable detection, often caused by tissue dilution or low abundance. White indicates no signal detection. STS cannot detect deep dermal pathology, such as Th1 signatures and structural remodeling. Biopsy effectively captures these deep signals. Blood sampling captures the systemic inflammatory load. This stratified profile indicates that an integrated multimodal sampling framework is necessary for precise AD phenotyping.

Consequently, relying exclusively on conventional STS sampling affords only a narrow, superficial characterization of disease biology [35, 48]. To address this critical limitation in analytical depth and advance STS into a clinically robust platform, it is essential to combine it with high‐sensitivity multi‐omics strategies—which maximize molecular information recovery from low‐biomass specimens—and to cross‐validate results against gold‐standard methods including skin biopsies and peripheral blood profiling [36]. This integrative strategy elevates STS from a technically limited sampling approach to a holistic methodological bridge (Figure 3).

#### 1.3.5 Integrating Tape Stripping and Skin Biopsies: From Epidermis to Dermis

A comprehensive understanding of AD requires the integration of the complementary strengths of different sampling methods. Skin biopsies provide detailed information on deep tissue structures and molecular changes within the dermis, whereas STS focuses on the epidermis, offering non‐invasive, repeatable access to its dynamic alterations. Therefore, discrepancies between biopsy and STS results should be interpreted not as methodological conflicts but as insights into layered pathological mechanisms.

Several RNA sequencing studies have directly compared tape strips and biopsies in AD patients, highlighting their complementary value [28, 30]. Tape strips are particularly sensitive to epidermal features, including pruritus‐associated genes (e.g., IL‐31), innate immune activation, and defects in terminal keratinocyte differentiation. In contrast, biopsies more accurately capture dermal cytokine dynamics, the Th22 inflammatory response, and tissue‐level abnormalities such as epidermal hyperplasia [35]. This differential sensitivity provides a strategic entry point for in‐depth mechanistic investigation.

For example, high Th1 immune signaling is predominantly observed in adult AD and is largely undetectable in tape strip samples. Further analysis revealed that Th1 signals originate primarily from the deep dermis, explaining STS detection limitations. This raises a critical question: does elevated Th1 expression in adult AD reflect an age‐related change, or is it a chronic disease phenotype emerging from systemic immune dysregulation in adulthood [16]?

The difference between deep tissue and superficial epidermis also highlights a more fundamental issue: does a molecule's potential correspond to its actual function? This discrepancy is particularly evident at the transcript and protein levels. A comparison study within the same individuals has demonstrated a lack of correlation between cytokine mRNA levels (potential) in biopsies and corresponding protein levels (function) in tape strips. These findings likely reflect complex post‐transcriptional and translational regulation, accounting for differences between full‐thickness gene transcripts and epidermal protein profiles [35].

These seemingly contradictory findings precisely highlight the unique insights offered by STS and emphasize the value of integrating multiple technologies. Combining STS with biopsies allows construction of a complete molecular map from the epidermis to the dermis, enabling a deeper analysis of the complex pathophysiological mechanisms underlying AD.

#### 1.3.6 Integrating Tape Stripping and Blood Analysis: Linking Local and Systemic Inflammation

Combining STS with blood analysis is essential for an integrated assessment of local and systemic inflammation in AD. While blood samples capture systemic immune status, they often fail to reflect specific pathological changes occurring within the skin. STS, in contrast, is particularly sensitive to innate pro‐inflammatory cytokines, T‐cell–recruiting chemokines, and tissue repair proteins, whereas serum better reflects systemic immune activity, including key T‐cell‐derived cytokines such as IL‐4 and IL‐13 [36].

This complementary approach has significant clinical value. In patients in remission, blood tests may reveal residual minimal persistent inflammation, whereas NMF levels measured by STS indicate whether the local epidermal barrier has been fully restored. Together, these measurements provide a more comprehensive assessment of recurrence risk [38]. Studies in infants further demonstrate that local corticosteroid treatment not only ameliorates epidermal inflammation, as detected by STS, but also normalizes systemic Th2 biomarkers in blood, supporting the concept that the skin is a major source of systemic inflammatory cytokines [20, 29].

#### 1.3.7 Multimodal Integration and Microbiome Insights: Broadening the Research Landscape

Beyond integration with blood analysis, STS serves as a central hub for molecular data, which can be combined with other non‐invasive skin assessment techniques to achieve multi‐modal data fusion. Combining molecular data from tape strips with physiological indicators, like TEWL and skin conductance, as well as imaging modalities like reflectance confocal microscopy, enables cross‐validation and enrichment of findings across multiple perspectives [6, 19, 42]. For example, combining tape strip proteomic data with TEWL measurements has identified protein clusters strongly correlated with barrier function, providing a precise foundation for the diagnosis, monitoring, and treatment of AD [55].

In microbiome research, STS has similarly advanced our understanding. Integrated RNA sequencing and lipidomics enable simultaneous characterization of microbial communities and lipid composition in AD skin [59], revealing the critical role of commensal microbiota in regulating ceramide metabolism and opening new avenues for investigating microbe‐lipid interactions [8, 9, 57]. Studies have also demonstrated that the natural variation in microbial diversity across different skin habitats—dry, moist, and sebaceous—is diminished in AD, indicating that the disease state overrides intrinsic environmental influences [69].

Importantly, metagenomic analyses reveal microbial functional potential (DNA), but metatranscriptomics is required to capture actual activity (RNA). Significant discrepancies are observed: abundant bacteria such as *Cutibacterium acnes* can exhibit low transcriptional activity, whereas larger eukaryotes like Malassezia may exert disproportionate functional effects [69]. RNA‐based analysis is therefore crucial to distinguish microbial presence from activity and to identify key pathogenic contributors in a compromised skin barrier [70].

Variability in STS data should be interpreted not as a limitation but as a source of mechanistic insight. The spatial heterogeneity between STS and biopsies reflects the stratified pathology of AD rather than methodological conflict: STS sensitively captures superficial epidermal processes, while biopsies reveal deeper dermal signals, highlighting their complementarity in constructing a complete molecular map of the skin [35].

Discrepancies within tape strip analyses at different molecular levels are similarly informative. For instance, the core Th2 cytokine IL‐13 shows consistent transcript upregulation in RNA sequencing, yet the corresponding protein is often difficult to detect [27, 30, 66]. Such discrepancies further highlight the added value of multi‐omics integration, as exemplified by TARC/CCL17 [19, 49, 61, 62]. While elevated mRNA transcripts captured by STS as early as 2 months of age serve as powerful biomarkers for predicting future AD onset [62], protein levels of the same chemokine are often more reflective of real‐time clinical severity and dynamic treatment response to systemic therapies [49, 61]. This complementarity demonstrates that transcriptomics and proteomics provide non‐redundant, synergistic layers of information—molecular potential versus biological function—which are essential for longitudinal, multi‐dimensional molecular phenotyping in AD.

Furthermore, the heterogeneity between local STS findings and systemic blood biomarkers further emphasizes STS's role as a bridge. STS can detect subclinical inflammation in the skin even when systemic markers are normal, supporting the concept of the skin as a primary source of systemic cytokines and capturing the local‐to‐systemic cascade [37]. This approach also reveals temporal heterogeneity, showing that different biomarkers are predictive for disease risk versus prognosis [60]. Investigating multi‐dimensional heterogeneity across spatial, molecular, systemic, and temporal domains therefore provides a more complete, dynamic, and nuanced understanding of AD pathophysiology.

### 1.4 Challenges and Perspectives

#### 1.4.1 Translational Challenges in STS‐Based Multi‐Omics

Despite the transformative insights afforded by combining STS with multi‐omics profiling, a substantial validation gap remains between exploratory biomarker discovery and clinical deployment. Several critical challenges must be addressed before these multidimensional datasets can inform routine clinical practice.

First, although state‐of‐the‐art multi‐omics sequencing partially compensates for the low biomass of STS samples, the minute quantities of starting material amplify technical challenges and yield large, high‐dimensional datasets that pose substantial bioinformatic hurdles. Advanced analytical pipelines and open‐access databases are urgently needed to harmonize data across heterogeneous populations and to define conserved core regulatory networks in AD pathogenesis.

Second, rigorous analytical validation represents a key translational bottleneck for STS to advance from an exploratory research tool to a clinically validated laboratory assay. Translating multi‐omics discoveries into affordable, rapid point‐of‐care platforms requires stringent, multicenter standard operating procedures (SOPs) that explicitly mitigate operator‐dependent variability—such as differences in applied pressure and stripping velocity—as well as anatomical site‐specific heterogeneity. Standardized normalization strategies are also essential to adjust for inherent disparities in stratum corneum sample yield and to ensure robust data reproducibility across diverse clinical populations [42].

Finally, generating comprehensive multi‐omics profiles demands a critical reassessment of the term “non‐invasive” in a clinical context. Although shallow STS is truly non‐invasive and well‐tolerated, deep‐coverage protocols required for full omics characterization—often involving up to 35 sequential strips—can fully deplete the stratum corneum, occasionally eliciting transient mild erythema and temporarily impairing local barrier integrity. Future clinical applications must therefore balance the molecular depth required for high‐quality omics analysis against patient comfort and acceptability. Successfully navigating these bioinformatic, regulatory, and operational challenges will ultimately determine the translation of STS into routine precision dermatologic care.

#### 1.4.2 Future Directions and Clinical Translation

To address the bioinformatic and regulatory hurdles outlined above, future efforts should prioritize advanced analytical pipelines tailored for dimensionality reduction. Open‐access, STS‐specific databases will facilitate cross‐study data harmonization, sharing, and re‐analysis. In parallel, network biology approaches that delineate host gene–protein–lipid–microbe interactions may reveal conserved core regulatory networks underlying AD pathogenesis, offering novel insights for targeted therapeutic development. Once universal standardization is achieved, translating these multi‐omics findings into cost‐effective, rapid point‐of‐care platforms will mark a critical milestone toward early diagnosis and personalized treatment of atopic dermatitis. Moreover, large‐scale, longitudinal STS cohorts will be vital to delineate its clinical utility in predicting the atopic march and monitoring the progression of systemic comorbidities. Comparative studies evaluating STS against other non‐invasive epidermal sampling approaches—such as sebum collection and skin swabbing—across key parameters including molecular yield and omics compatibility would further define its methodological niche.

Beyond RNAs, proteins, and lipids, STS enables interrogation of additional novel biomolecule classes. For instance, adhesive sebum‐capturing tapes have been used to profile skin hormones [52], opening new avenues for investigating local endocrine mediators in inflammatory skin disorders.

Ultimately, the clinical and translational utility of STS extends well beyond atopic dermatitis. The technique has been successfully applied to psoriasis [40, 71], juvenile dermatomyositis [44], hidradenitis suppurativa [72], and seborrheic dermatitis [73], uncovering distinct molecular signatures across these conditions. Future investigations may leverage STS to compare molecular profiles across diverse inflammatory skin diseases, identify shared pathological pathways and disease‐specific biomarkers, and foster a more integrated understanding of inflammatory skin disorders.

### 1.5 Conclusion

As a chronic, relapsing inflammatory skin disease, the management of AD and the assessment of treatment response rely on longitudinal molecular data. However, traditional sampling methods are invasive, often painful, and may leave scars, limiting patient compliance, particularly in infants and young children, and creating a bottleneck for clinical research.

In this context, STS represents more than a technical refinement; it offers a precise lens for investigating the epidermal compartment of AD. In this narrative review, we synthesize the STS landscape, highlighting how multi‐layered normalization—from physical pressure to bioinformatic calibration—ensures the robustness of molecular findings. By enabling minimally invasive, longitudinal sampling, STS captures the dynamic molecular trajectory of the skin barrier and innate immune responses without the confounding signals of the deeper dermis.

More importantly, STS exemplifies a conceptual shift toward “methodological multi‐omics,” serving as a critical epidermal bridge. A holistic understanding of AD emerges not from relying on a single technique, but from integrating complementary methods: STS captures epidermal dynamics, biopsies reveal deep tissue pathology, and blood analysis reflects systemic inflammation. This multi‐dimensional framework provides insights unattainable by any single approach and positions STS as a cornerstone of integrated research strategies, essential for advancing precision medicine in AD.

In summary, STS is more than a novel sampling method; it is a conceptual bridge linking basic research and clinical practice, facilitating the implementation of precision medicine. While challenges remain in standardization, data integration, and bioinformatics, the prospects are promising. Future efforts should focus on establishing robust standard operating procedures, developing advanced tools for multi‐omics data integration, and accelerating the clinical translation of findings. By complementing biopsies and blood‐based analyses, STS will continue to strengthen this bridge, ultimately advancing precision medicine for AD and improving the health and quality of life of millions of patients worldwide.

## Author Contributions

**Ziyuan Tian:** writing – original draft, writing – review and editing, investigation, conceptualization, methodology, visualization. **Ke Xue:** writing – review and editing, supervision, project administration, funding acquisition. **Yong Cui:** writing – review and editing, supervision, project administration, funding acquisition.

## Funding

This work was supported by the Key Program of the National Natural Science Foundation of China (Grant No. 82330097), the National Natural Science Foundation of China (Grant No. 82304013), and the National High Level Hospital Clinical Research Funding of China‐Japan Friendship Hospital (Featured Disciplines and Featured Technologies Project; Grant No. 2024‐NHLHCRF‐TS‐01).

## Ethics Statement

The authors have nothing to report.

## Consent

The authors have nothing to report.

## Conflicts of Interest

The authors declare no conflicts of interest.

## Data Availability

Data sharing not applicable to this article as no datasets were generated or analysed during the current study.
